# Drug-Dependent Enhancement of Blood–Brain Barrier Permeation by Polysorbate 80 Minor Components

**DOI:** 10.3390/pharmaceutics17121572

**Published:** 2025-12-05

**Authors:** Xiaofeng Wang, Jue Wang, Xia Zhao, Langui Xie, Rui Yang, Chunmeng Sun, Jiasheng Tu, Huimin Sun

**Affiliations:** 1 National Institutes for Food and Drug Control, 2 Tiantan Xili, Dongcheng District, Beijing 100050, China; 2 Center for Research Development and Evaluation of Pharmaceutical Excipients and Generic Drugs, Department of Pharmaceutics, School of Pharmacy, China Pharmaceutical University, 24 Tong Jia Xiang, Nanjing 210009, China

**Keywords:** polysorbate 80, polyoxyethylene sorbitan monooleate, polyoxyethylene isosorbide monooleate, polyoxyethylene sorbitan dioleate, polyethylene glycol/polyoxyethylene sorbitan/polyoxyethylene isosorbide mixture, blood–brain barrier

## Abstract

**Background/Objectives**: Polysorbate 80 (PS80), a complex surfactant mixture, is widely recognized for its ability to enhance drug permeation across the blood–brain barrier (BBB). While this effect is generally attributed to the combined actions of its components, the specific contribution and potential selectivity of individual minor components remain poorly understood. This study therefore aimed to isolate and compare the primary minor components of PS80 to determine whether they uniformly enhance BBB permeation or exhibit drug-specific functions. **Methods**: In this research, four primary minor components of PS80—polyoxyethylene sorbitan monooleate (PSM), polyoxyethylene isosorbide monooleate (PIM), polyoxyethylene sorbitan dioleate (PSD), and a polyethylene glycol/polyoxyethylene sorbitan/polyoxyethylene isosorbide mixture (PEG/PS/PI mixture)—were isolated using preparative liquid-phase chromatography. Drug-loaded formulations were then prepared using the solvent evaporation method incorporating five model drugs: 1,1′-dioctadecyl-3,3,3′,3′-tetramethylindotricarbocyanine iodide (DiR, MW = 1013.39 Da), donepezil (MW = 379.49 Da), nimodipine (MW = 418.44 Da), chlorogenic acid (MW = 354.31 Da), and paclitaxel (MW = 853.92 Da). The permeability of these formulations across the BBB was evaluated in BALB/c mice after intravenous administration. Brain distribution of the lipophilic dye DiR was assessed using fluorescence imaging, whereas brain homogenate concentrations of therapeutic drugs were quantified by UPLC-MS/MS. **Results**: Results revealed that the enhancement of brain delivery was dependent on both the specific minor component and the drug. The PEG/PS/PI mixture specially enhanced the brain homogenate concentration of donepezil to 11.8 ± 1.2 ng/mL, representing a 6.9-fold enhancement, while PIM micelles increased the delivery of DiR, donepezil, and nimodipine. In contrast, PSM and PSD micelles improved transport of only DiR and donepezil. The broad performance of PIM suggests a more flexible formulation—a hypothesis that warrants further validation. Conversely, none of the different minor components enhanced the delivery of chlorogenic acid or paclitaxel, underscoring the critical role of specific drug–component interactions. **Conclusions**: This component-resolved insight challenges the conventional perception of PS80 and provides a rational framework for engineering precision brain-targeted delivery systems by selecting functional minor components.

## 1. Introduction

Central nervous system (CNS) disorders, such as Alzheimer’s disease and gliomas, are significant causes of global disability and mortality [1,2,3]. The drug development success rate for CNS diseases remains strikingly low, with challenges arising from both the complexity of pathologies and the formidable presence of the blood–brain barrier (BBB) [4,5]. The BBB, a highly selective and semi-permeable barrier formed by the endothelial cells of the CNS microvasculature, plays a critical role in maintaining brain homeostasis [6,7]. Tight junctions between endothelial cells restrict the passage of most substances, allowing primarily the passive diffusion of lipid-soluble molecules with molecular weights below 400–600 Da [8,9,10]. Consequently, the BBB excludes more than 98% of small-molecule drugs and nearly all large-molecule therapeutics [11]. This barrier not only limits the treatment options for CNS disorders but also poses challenges for drugs that do cross it, as low targeting specificity can cause off-target side effects and may require higher doses, which can also lead to significant peripheral side effects [12]. To address these limitations, innovative drug delivery formulations capable of crossing the BBB and enhancing brain targeting are urgently needed. This is crucial not only to expand the range of potential drug candidates but also to achieve improved therapeutic outcomes for CNS disorders.

In recent years, various strategies for drug delivery across the BBB have been developed and evaluated, encompassing both invasive approaches, such as focused ultrasound and intracerebral injection, and non-invasive methods, including receptor-mediated transport and nano-based delivery systems [13,14,15]. Among the latter, nanoparticles and nanomicelles have shown significant potential. These nanocarriers facilitate brain targeting via various mechanisms, including adsorption-mediated endocytosis, thereby enhancing drug accumulation in the brain [16,17,18].

Polysorbate 80 (PS80) has been widely used as a surface modifier for nanoparticles, enhancing colloidal stability and promoting brain targeting. This effect is primarily attributed to the adsorption of apolipoprotein E onto the nanoparticle surface, which enables receptor-mediated transport across the BBB [19,20]. The performance of this strategy is well-established. For instance, Somasree Ray et al. [21] conducted in vivo biodistribution studies using Wistar rats and demonstrated that PS80-coated chitosan nanoparticles delivered significantly higher concentrations of ropinirole hydrochloride to the brain of Wistar rats than uncoated nanoparticles, while reducing accumulation in peripheral organs. Similarly, Andrea Joseph et al. [22] reported a 19-fold increase in brain uptake of PS80-coated poly(lactic-co-glycolic acid)-poly(ethylene glycol) nanoparticles after intravenous administration, compared to unmodified controls.

Beyond its role as a coating agent, PS80 is a surfactant capable of self-assembling into micelles—a class of nanocarriers valued for their inherent self-assembly properties and ease of preparation [23]. Various micellar formulations have demonstrated effective BBB penetration, underscoring the potential of micelles as brain delivery platforms. For example, a 2020 study [24] developed a novel micelle-based drug delivery system consisting of PLGA-lyso-monosialotetrahexosylganglioside (GM1) loaded with doxorubicin. In vivo experiments confirmed that these micelles effectively crossed the BBB and specifically accumulated in the brain, demonstrating their potential for targeted brain drug delivery. Another study [25] designed a disulfide bond-coupled prodrug polymer combining camptothecin and polyethylene glycol (PEG), further modified with the iRGD peptide for enhanced targeting. Drug-loaded micelles were prepared via self-assembly, and the system exhibited excellent BBB penetration and glioma-targeting ability both in vitro and in vivo.

However, PS80 is not a single molecule but a complex mixture. It comprises fatty acid esters (mainly oleic acid) formed by the partial esterification of ethoxylated derivatives of 1 mol sorbitol/isosorbitol with approximately 20 mol ethylene oxide [26]. Our previous analysis using ultra-performance liquid chromatography tandem quadrupole time-of-flight mass spectrometry (UPLC-Q-TOF-MS) identified nine distinct minor components in PS80 [27]. These minor components can be broadly categorized based on their solubility profiles. The highly water-soluble minor component is the polyethylene glycol/polyoxyethylene sorbitan/polyoxyethylene isosorbide mixture (PEG/PS/PI mixture). In contrast, the highly fat-soluble minor components include polyoxyethylene sorbitan monooleate (PSM), polyoxyethylene isosorbide monooleate (PIM), and polyoxyethylene sorbitan dioleate (PSD), among others.

Critically, while the overall efficacy of PS80 is recognized, its intrinsic compositional complexity has been treated as a “black box.” The individual contributions of these specific minor components to BBB shuttling—and whether they function uniformly or exhibit drug-dependent selectivity—remain entirely unclear. This knowledge gap limits the rational design of brain-targeted formulations.

To address this, we evaluated the functional performance of PS80’s principal minor components using a micelle-based delivery model. This approach allows the intrinsic properties of each component to be assessed directly, free from the variables introduced by a nanoparticle core. Our work is the first to deconstruct this overall effect by directly linking specific minor components to enhanced delivery for particular drugs, thereby revealing functional specificity that has previously been overlooked.

**Figure 1:**
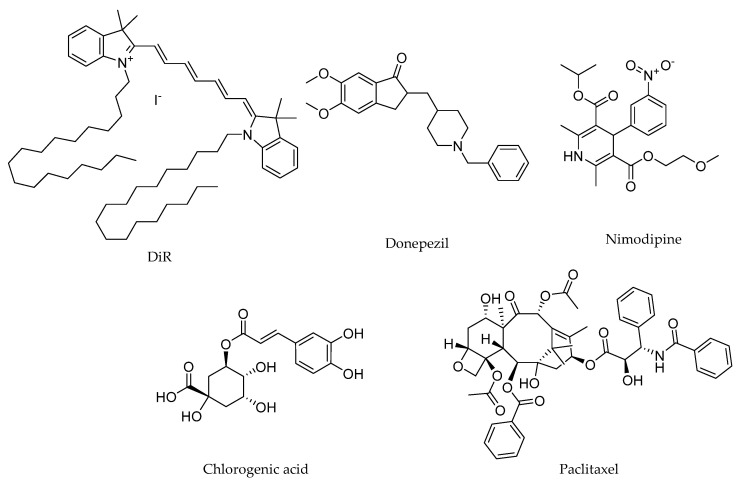
The chemical structure of the model drugs.

The drugs investigated in this study included 1,1′-dioctadecyl-3,3,3′,3′-tetramethylindotricarbocyanine iodide (DiR), donepezil, nimodipine, chlorogenic acid, and paclitaxel. DiR is a lipophilic fluorescent dye commonly used to stain cell membranes and other fat-soluble biological structures, and it serves as a reliable marker for in vivo imaging and tracking studies [28,29,30]. Donepezil is a reversible acetylcholinesterase inhibitor widely used for the symptomatic treatment of mild-to-moderate Alzheimer’s disease by enhancing cholinergic neurotransmission [31]. Nimodipine, a dihydropyridine calcium channel blocker, has been studied for its potential to improve cerebral blood flow and prevent cognitive decline in patients with Alzheimer’s disease and subarachnoid hemorrhage [32,33]. Chlorogenic acid, a prominent dietary polyphenol, exhibits neuroprotective properties through its antioxidative and anti-inflammatory effects [34]. Paclitaxel, a chemotherapeutic agent primarily used for treating solid tumors, has shown potential in preclinical models for targeting glioblastoma, though its clinical application is limited by poor BBB permeability [35,36]. These drugs were selected to represent a broad spectrum of physicochemical and pharmacokinetic properties (Table 1), which dictate their distinct brain uptake mechanisms. Donepezil crosses the BBB primarily via passive diffusion, owing to its molecular weight (<400 Da) and lipophilicity (Log P ~4) [37]. In contrast, the other four drugs are hindered by specific barriers: DiR is effectively excluded from BBB penetration due to its excessively large molecular weight (>1000 Da), which precludes passive diffusion, despite its high lipophilicity. Nimodipine and paclitaxel are substrates for P-glycoprotein (P-gp)-mediated efflux [38,39], despite their moderate lipophilicity. Chlorogenic acid’s penetration is limited by its high hydrophilicity (Log P < 0) and high hydrogen-bonding capacity, which are unfavorable for passive diffusion. As illustrated in Figure 1, the chemical structures of these drugs reflect their diverse properties. These characteristics make them ideal candidates for systematically evaluating the role of PS80 minor components in enhancing drug delivery across the BBB.

To address the unresolved question of component-specific functionality, this study aimed to systematically deconstruct the BBB permeation-enhancement effect of PS80 by evaluating its individual minor components. The work had two primary objectives: first, to isolate the principal minor components of PS80—namely PSM, PIM, PSD, and the PEG/PS/PI mixture; and second, to evaluate their performance in enhancing brain delivery in BALB/c mice. This evaluation involved assessing DiR distribution via fluorescence imaging at 60 min after intravenous administration and quantifying the brain homogenate concentrations of four therapeutic drugs (donepezil, nimodipine, chlorogenic acid, and paclitaxel) via UPLC-MS/MS at 10 min after intravenous administration.

We hypothesized that the BBB permeation-enhancement capability of PS80 is not a unified function of the mixture as a whole, but rather stems from the distinct, drug-dependent activities of its specific minor components. Consequently, the optimal component for facilitating drug transport across the BBB would vary with the specific drug candidate. The experimental approach to test this hypothesis is described below.

## 2. Materials and Methods

### 2.1. Reagents and Materials

PS80 was purchased from Nanjing Weir Chemical Co., Ltd. (Nanjing, China). DiR was obtained from Beijing Zhongsheng Ruitai Science and Technology Co., Ltd. (Beijing, China). Donepezil and nimodipine were sourced from Hubei Jusheng Science and Technology Co., Ltd. (Tianmen, China) and Aladdin (Shanghai, China), respectively. Chlorogenic acid was provided by the National Institutes for Food and Drug Control (NIFDC, Beijing, China). Paclitaxel and pyrene were obtained from Sigma-Aldrich (St. Louis, MO, USA). Saline was supplied by Shijiazhuang Shiyao Co., Ltd. (Shijiazhuang, China). Tetrahydrofuran (THF), methanol, isopropanol, acetonitrile, and ethanol were of chromatography grade, while ammonium acetate and formic acid were of mass spectrometry grade. Deionized water was prepared in-house and used for all experiments.

### 2.2. Animal Use and Ethics Statement

BALB/c mice (approximately 20 g, mixed sex), were obtained from a licensed animal supplier and housed in the specific pathogen-free facility of NIFDC. The animals were maintained under controlled conditions (temperature: 22 ± 2 °C, humidity: 50% ± 10%, 12 h/12 h light/dark cycle) with free access to food and water. All animal experiments were approved by the Institutional Animal Care and Use Committee of NIFDC on 17 August 2016 (ethics approval number: 2016(B)009) and conducted in compliance with the National Guidelines for the Care and Use of Laboratory Animals.

### 2.3. HPLC Systems for PS80 Separation

PS80 was isolated according to our previously established laboratory method [27]. Briefly, PS80 was accurately weighed, dissolved in methanol, and diluted to a concentration of approximately 1 mg/mL. The solution was then analyzed using a high-performance liquid chromatography (HPLC) system (Agilent 1260, Agilent Technologies, Santa Clara, CA, USA) equipped with an evaporative light scattering detector (ELSD) (Agilent 1260 Infinity II, Agilent Technologies, Santa Clara, CA, USA). Separation was achieved on an Agilent Eclipse XDB-C18 column (4.6 × 150 mm, 5 μm) maintained at 30 °C. The mobile phase consisted of (A) THF and (B) methanol, with the following gradient program: 0–5 min, 100% to 90% B; 5.0–19 min, 90% to 20% B; 19–22 min, 20% to 100% B; and 22–25 min, 100% B. The flow rate was 1.0 mL/min, and the injection volume was 20 µL. The ELSD parameters were set as follows: drift tube temperature, 100 °C; gain 1; and nitrogen flow rate, 1.6 L/min.

### 2.4. UPLC-Q-TOF-MS Systems for PS80 Minor Components Structural Confirmation

Structural confirmation of PS80 minor components was performed using a previously established UPLC-Q-TOF-MS method [27]. The analysis was conducted on a UPLC-Q-TOF-MS system (Agilent 1290-6550, Agilent Technologies, Santa Clara, CA, USA) equipped with Agilent MassHunter Workstation (version B.08.00). The mobile phase was modified from the HPLC method (Section 2.3) by replacing THF with isopropanol and adding 0.1% formic acid to improve ionization efficiency, and all other chromatographic conditions remained unchanged. The total ion chromatogram and mass spectra were extracted under the following mass spectrometry conditions: ion mode, Jet Stream ESI positive; drying gas temperature, 300 °C; drying gas flow, 8 L/min; nebulizer pressure, 40 psi; sheath gas temperature, 350 °C; sheath gas flow, 11 L/min; capillary voltage, 3500 V; fragmentor voltage, 400 V; and mass range, 100–3000 *m*/*z*. A personal compound database and library (PCDL) for PS80 was constructed based on its raw materials and production processes. Compound identification was automated using the Find by Formula algorithm within the MassHunter software, which matched accurate mass data against the custom PCDL.

### 2.5. Chromatography Systems for Four Minor Components Collection

The four minor components—PEG/PS/PI mixture, PSM, PIM, and PSD—were collected using a previously established preparative HPLC method [27]. Briefly, an appropriate amount of PS80 was accurately weighed, dissolved in THF, and diluted to a concentration of 500 mg/mL. The separation was performed on a Waters Auto Purification System 2767 (Waters Corporation, Alameda, CA, USA), equipped with a Gemini-NX column (150 × 21.2 mm, 5 μm) and an ELSD. The mobile phases consisted of (A) methanol–water (95:5, *v*/*v*) and (B) THF, with the following gradient program: 0–15min, 0% to 50% B; 15–15.1min, 50% to 100% B; 15.1–17min, 100%B; 17–17.1min, 100% to 0% B; and 17.1–19min, 0% B. The flow rate was 25 mL/min, and the injection volume was 800 μL. Fraction collection was triggered by the ELSD signal using the following settings: drift tube temperature, 60 °C; and carrier gas pressure, 60 psi.

### 2.6. Determination of the Critical Micelle Concentration of PS80 Minor Components

The critical micelle concentration (CMC) of each isolated PS80 minor component was determined using the pyrene fluorescence probe method. In this method, the intensity ratio of the first (I_1_, 374 nm) to the third (I_3_, 384 nm) vibrational peaks in pyrene’s fluorescence emission spectrum is highly sensitive to environmental polarity. A sharp decrease in the I_1_/I_3_ ratio indicates pyrene incorporation into the non-polar core of a micelle, signaling micelle formation. Briefly, a series of eighteen solutions with minor component concentrations ranging from 0.1 to 35 μg/mL were prepared, each containing a fixed mass of pyrene (0.6 μg). Fluorescence spectra were recorded on a fluorescence spectrophotometer (RF-5301PC, Shimadzu corporation, Kyoto, Japan) with an excitation wavelength of 336nm. The I_1_/I_3_ ratio was plotted against the logarithm of the surfactant concentration, and the data were fitted to a Boltzmann sigmoidal curve. The CMC value was defined as the inflection point of this fitted curve.

### 2.7. Preparation and Physical Characterization of Drug-Loaded Formulations

Drug-loaded formulations were prepared using the solvent evaporation method [45]. Specifically, for each formulation, a drug stock solution in ethanol was transferred into a round-bottom flask. To this, 0.2 mL of ethanol, 0.5 mL of a 10.0 mg/mL minor component saline solution, and 0.5 mL of saline were added. Ethanol was removed by rotary evaporation using a rotary evaporator set at 35 °C until the solvent was completely evaporated, yielding drug-loaded formulations. Each 1 mL of the resulting solutions contained 0.3 mg of DiR, 0.3 mg of donepezil, 0.3 mg of nimodipine, and 0.3 mg of chlorogenic acid, or 0.1 mg of paclitaxel, respectively, and was used as the experimental drug-loaded formulation group. For the vehicle control group, solutions of 0.3 mg/mL DiR, donepezil, nimodipine, and chlorogenic acid in ethanol and 0.1 mg/mL paclitaxel in ethanol were prepared to represent the unformulated drugs. This group represents the baseline performance of the free, unformulated drugs. Pure saline was employed as a blank control to establish the baseline for background signal in all subsequent drug quantification analyses.

The mean particle size and polydispersity index (PDI) were determined after diluting the drug-loaded formulations with saline to achieve a photon count rate within the optimal range (100–500 kcps) for dynamic light scattering (DLS) instrument using a Zetasizer Nano ZS (Malvern, UK). Zeta potential was measured on the same instrument on undiluted samples to preserve the native electrochemical environment. For morphology observation, an appropriate amount of each sample was dropped onto a copper grid, stained with 4% phosphotungstic acid for 1–2 min, and allowed to air dry. The morphology of the samples was observed using a transmission electron microscopy (TEM, JEM1200EX, JEOL, Tokyo, Japan) at appropriate magnifications.

### 2.8. Quantification of DiR in Mice Brain

A total of thirty 4-week-old BALB/c mice, comprising equal numbers of males and females, were randomly assigned to six groups (*n* = 5 per group). Each group received a single intravenous injection via the tail vein with one of the following: DiR-loaded formulations, DiR ethanol solution, or pure saline. The administered dose of DiR was set at 3 mg/kg. The in vivo distribution of DiR was monitored using an in vivo imaging system (IVIS Lumina Series III, Xenogen, Milford, MA, USA) at 5 min and 60 min post-injection. At the 60 min endpoint—chosen based on the imaging data that confirmed sustained brain retention—mice were euthanized via cervical dislocation, and brain tissues were harvested. The fluorescence intensity of DiR in the brain tissues was measured using the imaging system, with excitation and emission wavelengths set at 740 nm and 790 nm, respectively. One-way analysis of variance (ANOVA) was used to determine whether different minor components had a statistically significant effect on DiR transport across the BBB. A *p* < 0.05 was considered statistically significant.

### 2.9. Quantification of Donepezil, Nimodipine, Chlorogenic Acid, and Paclitaxel in Mice Brain

A total of one hundred and twenty 4-week-old BALB/c mice, comprising equal numbers of males and females, were randomly assigned to 24 groups (*n* = 5 per group). Each group received a single intravenous injection via the tail vein with one of the following: drug-loaded formulations, ethanol solutions of the drugs, or pure saline. The dosage was set at 3 mg/kg. To capture the early distribution phase and sensitively compare initial brain uptake, a terminal time point of 10 min post-injection was selected. At this time, mice were euthanized via cervical dislocation. Brain tissues were carefully harvested, rinsed with saline to remove residual blood, gently blotted dry with sterile filter paper, and stored at −20 °C in sealed containers to prevent contamination or degradation.

For analysis, the brain tissues were weighed and homogenized in saline at a ratio of 2 mL saline per gram of tissue. The homogenate was vortexed thoroughly and centrifuged at 10,000 rpm for 15 min at 4 °C. Subsequently, 200 μL of the supernatant was transferred to a 1.5 mL microcentrifuge tube, mixed with 600 μL of methanol, vortexed, and centrifuged again under the same conditions. The resulting supernatant was transferred into a clean tube and evaporated to dryness under a nitrogen stream at room temperature. The residue was reconstituted in 300 μL of methanol, vortexed thoroughly, centrifuged, and the final supernatant was collected for ultra-performance liquid chromatography–tandem triple quadrupole mass spectrometry (UPLC-MS/MS) analysis. Quantification of drug concentrations in brain tissue homogenate (reported in ng/mL) was performed using the external standard method. Standard solutions were prepared by dissolving reference compounds in methanol and performing serial dilutions.

The UPLC-MS/MS analysis was conducted on an SHIMADZU 8050 system (Kyoto, Japan) equipped with a Kinetex C18 column (50 × 2.1 mm, 2.6 μm). The column temperature was maintained at 30 °C, with a flow rate of 0.3 mL/min. The mobile phase consisted of (A) 5 mM aqueous ammonium acetate and (B) acetonitrile, with the following gradient program: 0–1.5 min, 95% to 5% A; 1.5–2.0 min, 5% A; 2.0–2.6 min, 5% to 95% A; and 2.6–3.5 min, 95% A. The electrospray ionization source temperature was set at 300 °C with a voltage 3 kV. Data were acquired in multiple reaction monitoring (MRM) mode for both quantification and characterization of the analytes. The specific ionic reactions monitored were as follows:

A. Donepezil: characterization ions: *m*/*z* 380.3 → *m*/*z* 91.0, 243.1, and 362.2; quantification ion: *m*/*z* 91.0.

B. Nimodipine: characterization ions: *m*/*z* 419.2 → *m*/*z* 343.1 and 301.0; quantification ion: *m*/*z* 343.1.

C. Chlorogenic acid: characterization ions: *m*/*z* 353.2 → *m*/*z* 191.0 and 93.0; quantification ion: *m*/*z* 191.0.

D. Paclitaxel: characterization ions: *m*/*z* 854.6 → *m*/*z* 286.4 and 569.5; quantification ion: *m*/*z* 286.4.

All mass spectrometric parameters were optimized for maximum sensitivity and selectivity for each MRM analysis. The linear concentration ranges were as follows: donepezil, 1.0–100.0 ng/mL; nimodipine, 0.1–10.0 ng/mL; chlorogenic acid, 0.01–1.0 ng/mL; and paclitaxel, 10.0–100.0 ng/mL. All calibration curves exhibited excellent linearity, with correlation coefficients (r) greater than 0.99. The lower limit of quantification (LLOQ) for each analyte, defined as the lowest point on the calibration curve, was as follows: donepezil, 1.0 ng/mL; nimodipine, 0.1 ng/mL; chlorogenic acid, 0.01 ng/mL; and paclitaxel, 10.0 ng/mL.

Statistical analyses were selected based on the distribution of the data. For data satisfying parametric assumptions, a one-way ANOVA or Welch’s ANOVA was used. For non-normally distributed data or datasets containing non-quantifiable measurements, the Kruskal–Wallis H test was employed. When a significant overall difference was detected, it was further explored with respective post hoc tests. A *p* < 0.05 was considered statistically significant.

## 3. Results and Discussion

### 3.1. Structural Features and Purity of the Isolated PS80 Minor Components

**Figure 2:**
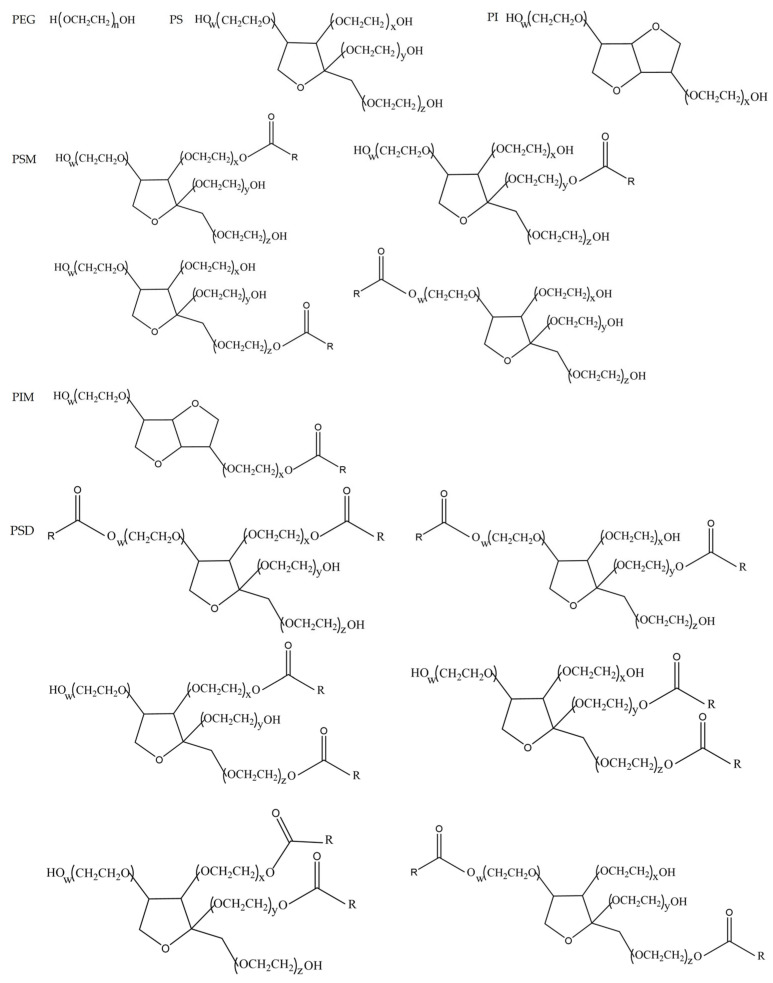
Chemical structural formulas of the four minor components. R denotes the oleate group.

Based on our previous work [27], PS80 has been chromatographically separated and its four principal minor components have been structurally identified. The purity of the isolated PEG/PS/PI mixture, PSM, PIM, and PSD was determined to be 100.0%, 95.3%, 95.4%, and 99.5%, respectively, using the peak area normalization method. The chemical structural formulas of these components are presented in Figure 2.

### 3.2. Micellization Potential of the PS80 Minor Components

The CMC of each isolated minor component was determined to evaluate their micellar self-assembly capability. The highly hydrophilic PEG/PS/PI mixture, which lacks a lipophilic domain, showed no detectable CMC, confirming its inability to form conventional micelles. Conversely, the amphiphilic components (PSM, PIM, PSD) exhibited low CMC values of 19.0 μg/mL, 6.8 μg/mL, and 6.6 μg/mL, respectively, confirming their inherent ability to form micelles at low concentrations. The distinct values among them are a direct reflection of their headgroup and tail chemistry: PSD, with two oleate chains, exhibits the strongest hydrophobic effect and thus the lowest CMC. Compared to PSM’s sorbitan headgroup, PIM’s isosorbide headgroup is less hydrophilic, which enhances the effective hydrophobic drive for self-assembly, resulting in a lower CMC. Collectively, these CMC results provide a physicochemical basis for their divergent self-assembly behaviors.

### 3.3. Physicochemical Properties of the Prepared Formulations

**Figure 3:**
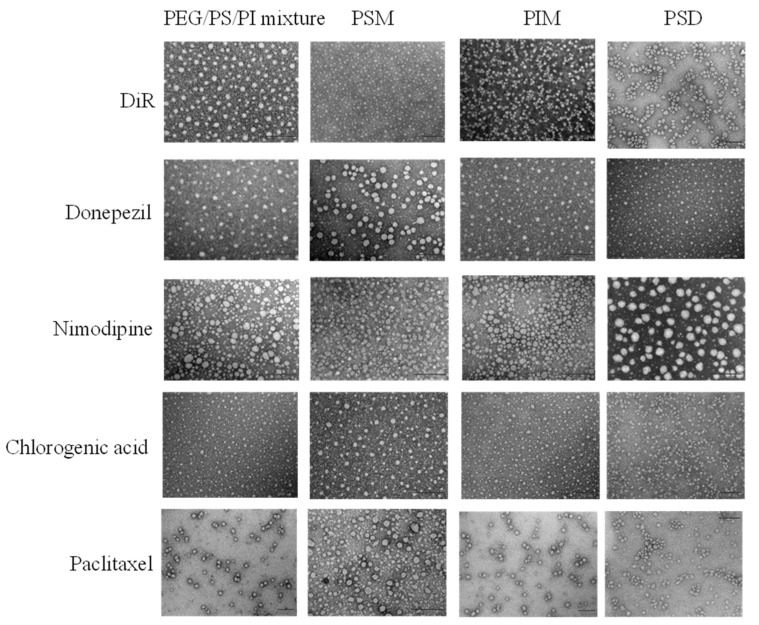
Morphology of drug-loaded formulations observed by TEM. Scale bars represent 200 nm for all samples.

Representative TEM images in Figure 3 show the spherical-like morphology of all the prepared formulations. However, TEM is insufficient to resolve the fundamental structural differences among these colloidal systems. To delineate these differences, the mean particle size, PDI, and Zeta potential of drug-loaded formulations prepared by the solvent evaporation method are presented in Table 2 and Table 3. Physicochemical properties confirmed the formation of diverse colloidal systems. While most formulations were nanoscale, a broad size distribution was observed—from the minimal micelles of PSD to the larger assemblies of PEG/PS/PI and the macroscopic aggregates of paclitaxel-loaded systems. Crucially, a near-neutral zeta potential is crucial for minimizing opsonization, thereby imparting the intrinsic stealth capability necessary for systemic delivery [46].

### 3.4. Drug-Dependent Enhancement of BBB Permeability by PS80 Minor Components

The ability of PS80-coated nanocarriers to enhance drug delivery to the brain is well-documented [17,18,19,20,21]. However, these prior studies have largely treated PS80 as a functionally uniform excipient. Our study challenges this view by systematically deconstructing PS80 into its constituent minor components and evaluating their individual contributions to BBB permeation.

Our study revealed that the BBB permeation-enhancement effect of PS80 is not uniform but exhibits remarkable component–drug specificity. This was established by evaluating the influence of PS80 minor components using five model drugs with diverse physicochemical properties. Notably, no drug-related signals were detected in pure saline-treated (blank control) animals, confirming the absence of analytical interference across all experiments.

**Figure 4:**
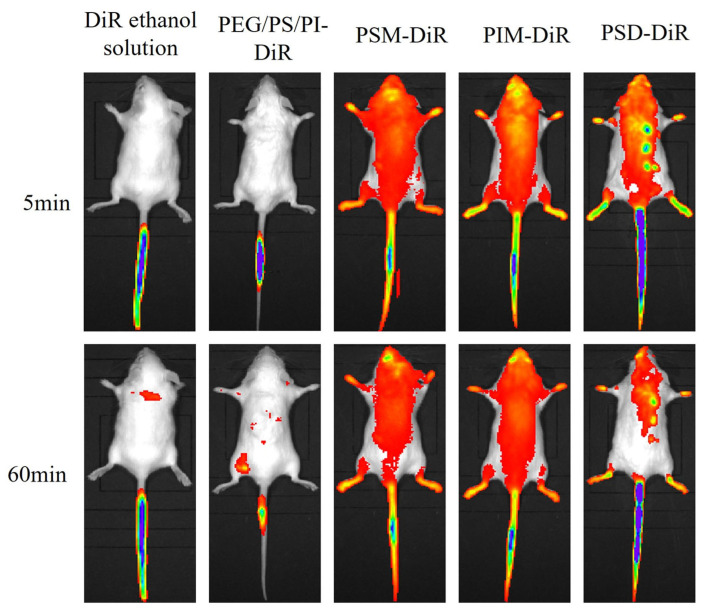
Distribution of DiR-loaded formulations of minor components in mice at 5 and 60 min post-injection.

**Figure 5:**
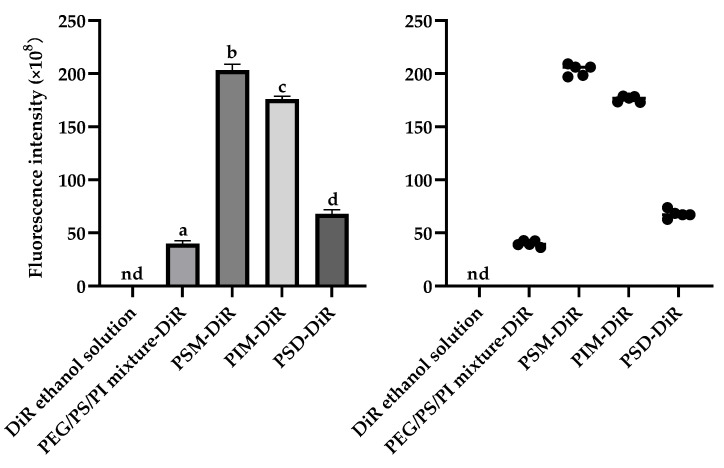
DiR fluorescence intensity in mouse brain tissues at 60 min post-injection. The column bar graph shows mean ± SD (*n* = 5). Significance was determined by one-way ANOVA with Tukey’s post hoc test for all pairwise comparisons. Bars not sharing a common lowercase letter (a, b, c, d) are significantly different. The scatter plot displays individual data points with lines representing median values. “nd” denotes non-detectable. The DiR ethanol solution was the vehicle control group.

For the lipophilic dye DiR, in vivo imaging revealed distinct distribution profiles (Figure 4). ANOVA revealed significant differences in brain delivery efficiency among the formulations (F (3,16) = 2158.25, *p* < 0.01). While free DiR in ethanol failed to achieve detectable fluorescence, all PS80 minor component formulations successfully delivered DiR to the brain, with fluorescence visible within 5 min and sustained for 60 min. The brain tissue fluorescence intensity at the 60 min endpoint (Figure 5) demonstrated that the ability of PS80-formulated DiR to reach the brain depended on the presence of specific minor components. The amphiphilic components (PSM, PIM, PSD) were vastly more efficacious, producing intense fluorescence. The PEG/PS/PI mixture, despite its success over the vehicle control (DiR ethanol solution), yielded only minimal delivery. This indicates that the amphiphilic components not only facilitate initial brain uptake but also significantly enhance and sustain the brain concentration of DiR over time. The delivery disparity likely arises from the nanostructures formed by the components. The components of PSM, PIM, and PSD assemble into stable micelles that efficiently encapsulate and protect DiR, enabling robust BBB transport and intense fluorescence. In contrast, the PEG/PS/PI mixture forms unstable assemblies with poor DiR retention, leading to payload leakage and minimal brain delivery.

**Figure 6:**
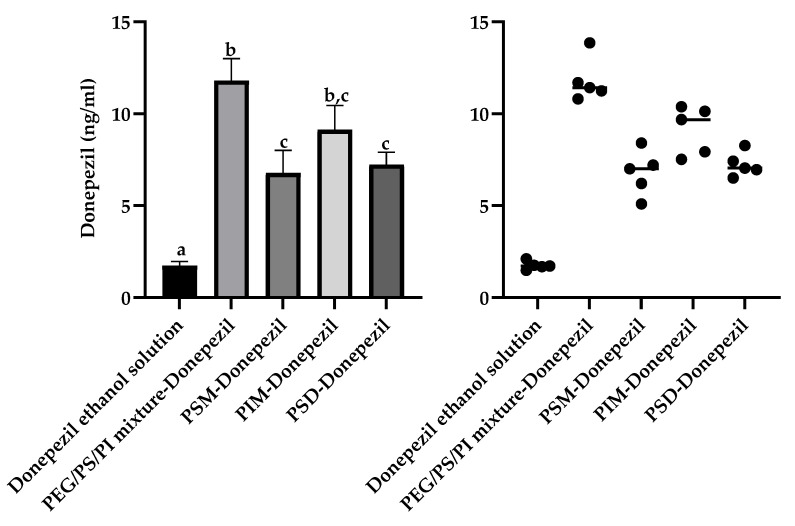
Accumulation concentration of donepezil in the mouse brain tissue homogenate. The column bar graph shows mean ± SD (n = 5). Significance was determined by Welch’s ANOVA followed by the Games–Howell post hoc test for all pairwise comparisons. Bars not sharing a common lowercase letter (a, b, c) are significantly different. The a-b difference demonstrates the potent brain delivery enhancement by the PEG/PS/PI mixture over the vehicle control. The b-c difference indicates that this mixture is a more effective formulation for donepezil than the PSM or PSD micelles. PIM-Donepezil group is labeled ‘b, c’ because it is not statistically different from groups labeled ‘b’ or ‘c’. The scatter plot displays individual data points with lines representing median values. The donepezil ethanol solution was the vehicle control group.

This drug-dependent enhancement profile was further elaborated using the therapeutic agents donepezil and nimodipine. For donepezil, a Welch’s ANOVA revealed that all minor component formulations significantly increased brain accumulation (W (4,8.7) = 160.0, *p* < 0.001) relative to the vehicle control (donepezil ethanol solution) (1.7 ± 0.2 ng/mL), but with a distinct enhancement hierarchy (Figure 6). The PEG/PS/PI mixture achieved the numerically highest brain homogenate concentration (11.8 ± 1.2 ng/mL, 6.9-fold), which was comparable to that of PIM micelles (9.1 ± 1.3 ng/mL, 5.4-fold). The PEG/PS/PI mixture significantly outperformed PSD micelles (7.2 ± 0.7 ng/mL, 4.2-fold) and PSM micelles (7.0 ± 1.2 ng/mL, 4.1-fold). The superior performance of the non-amphiphilic PEG/PS/PI mixture is consistent with a unique mechanism for this relatively hydrophilic drug, potentially involving stabilization through the formation of a soluble assembly. Such an interaction could enhance donepezil’s aqueous solubility and/or mitigate its recognition by efflux transporters like P-gp.

**Figure 7:**
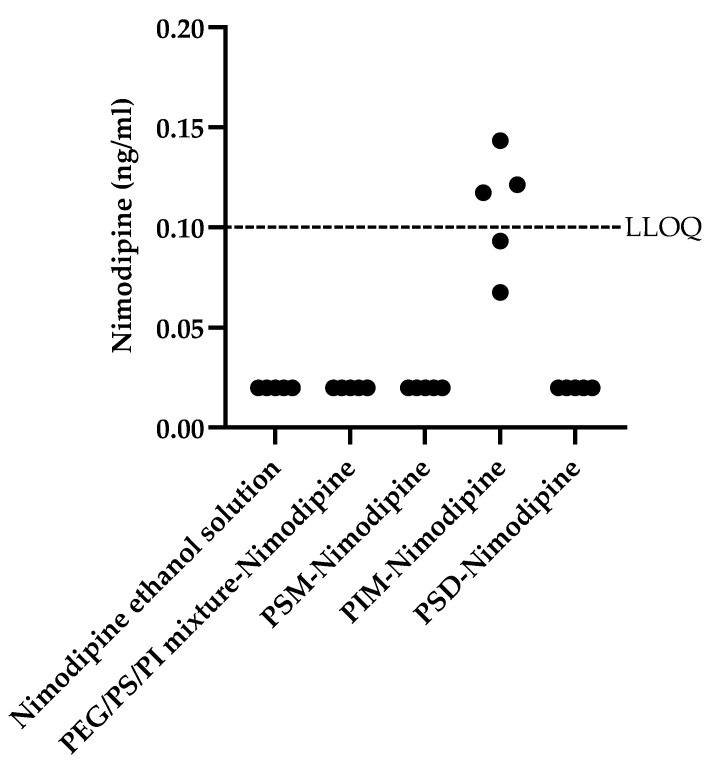
Accumulation concentration of nimodipine in the mouse brain tissue homogenate. The horizontal dashed line indicates the lower limit of quantification (LLOQ) for nimodipine (0.1 ng/mL). All measured values for the nimodipine ethanol solution (vehicle control group), PEG/PS/PI mixture, PSM, and PSD groups were below the LLOQ; these data points are plotted at the bottom of the graph for illustrative purposes. Data for the PIM group are presented as individual scatter points; values above the LLOQ line represent accurately quantifiable concentrations, while those below the line were detected but not quantifiable. The nimodipine ethanol solution was the vehicle control group.

In contrast to the broader enhancement profile of donepezil, nimodipine penetration was only enhanced by PIM micelles (Figure 7). Due to the fact that nimodipine concentrations for most formulations were below the LLOQ, standard parametric analysis was not appropriate. Therefore, a Kruskal–Wallis H test was conducted to assess differences in nimodipine brain homogenate concentrations across the five formulations. The test revealed a statistically significant effect, χ^2^ (4, N = 25) = 23.62, *p* < 0.001. Post hoc Dunn’s tests with a Bonferroni correction showed that the brain homogenate concentration in the PIM group was significantly higher than in the vehicle control group (nimodipine ethanol solution) (*p* < 0.01) and all other formulation groups (all *p* < 0.01). No significant differences were found among the vehicle control, PSM, PSD, and PEG/PS/PI groups. This remarkable selectivity underscores critical structure–function relationships. The unique performance of PIM could be attributed to its role as a more flexible formulation, a property perhaps related to its distinct chemical structure. The failure of the PEG/PS/PI mixture with nimodipine underscores a likely hydrophilic–lipophilic mismatch, preventing effective drug–component association.

Both chlorogenic acid and paclitaxel failed to cross the BBB with any formulation. The measured concentrations in all brain tissue homogenate samples were below the LLOQ. The LLOQ for chlorogenic acid was 0.01 ng/mL and for paclitaxel was 10.0 ng/mL. Although no precipitation was observed during preparation, chlorogenic acid failed to reach the brain with any formulation. This suggests that the minor components provided apparent solubility but could not achieve stable nano-encapsulation, presumably due to the drug’s extreme hydrophilicity and rigid structure. It is plausible that the drug rapidly dissociated from the carriers in the bloodstream and was cleared before reaching the BBB. The failure to deliver paclitaxel was due to macroscopic aggregation (4.5–30 μm), caused by its incompatibility with the PS80 minor components, which precluded the formation of stable, BBB-penetrating nanocarriers.

These results collectively establish that PS80’s permeation-enhancing effect is not universal but depends on highly specific component–drug interactions (Table 4). As summarized in the table, the non-amphiphilic PEG/PS/PI mixture enhanced BBB permeation of DiR and donepezil, but not nimodipine. While PSM and PSD micelles significantly improved transport of DiR and donepezil, they showed no effect on nimodipine. Notably, PIM micelles uniquely enhanced permeation of all three drugs—DiR, donepezil, and nimodipine. However, none of the components facilitated BBB transport of paclitaxel or chlorogenic acid.

Importantly, semi-quantitative analysis of PS80 from different manufacturers reveals substantial source-to-source variability in minor component distribution (Supplementary Table S1). For instance, the relative abundance of the PEG/PS/PI mixture varies nearly three-fold (15–44%), PIM varies over three-fold (5–16%), and PSD shows the greatest variation at seven-fold (4–28%). This compositional variability means that PS80 cannot be considered a functionally consistent excipient. The performance of PS80-based formulations depends critically on the specific product’s minor component profile. Our work establishes that functional performance is determined by both the ratio of these components and their specific compatibility with the drug candidate.

Having established the distinct functions of individual PS80 minor components, our future work will focus on deconvoluting their unique mechanisms. This shift from phenomenological observation to a component-specific investigation is crucial for building a predictive framework for rational carrier design. Key areas of inquiry are as follows:

First, deconvoluting the unique mechanisms of action for each minor component, utilizing time-course and full pharmacokinetic analyses to define the kinetics and specificity of BBB modulation.

Second, rational optimization of the most promising carriers, informed by a deeper understanding of their self-assembly (e.g., CMC under physiological stress) and their stability in biologically relevant environments.

Third, correlating the enhanced parenchymal drug levels demonstrated here with functional therapeutic outcomes in disease models, establishing the clinical potential of these systems.

These investigations will be vital to assess the translational potential of minor component-based delivery systems and to guide their development toward safe and effective clinical use.

## 4. Conclusions

This study systematically deconstructs the functionality of PS80, demonstrating that its brain-targeting capability emerges from highly specific, drug-dependent activities of its individual minor components rather than a unitary mechanism. This component–drug specificity was quantitatively underscored by the stark delivery disparities. For instance, the PIM formulation uniquely enhanced nimodipine brain concentration to a mean of 0.1 ng/mL, a significant increase over the vehicle control and the other formulations where levels were undetectable (<LLOQ), while the PEG/PS/PI mixture boosted donepezil accumulation by 6.9-fold, reaching a brain homogenate concentration of 11.8 ± 1.2 ng/mL, relative to the vehicle control (1.7 ± 0.2 ng/mL). The marked performance of the traditionally overlooked PEG/PS/PI mixture in enhancing donepezil delivery—potentially acting as a stabilizer—challenges its conventional classification as an inactive impurity. This performance is likely mediated by a PEGylation-like mechanism that prolongs circulation and enhances BBB penetration [47,48]. Meanwhile, the ability of PIM to enhance the delivery of DiR, donepezil, and nimodipine indicates that it is a more versatile formulation, capable of accommodating a wider range of drug properties. Crucially, the universal ineffectiveness of all components against chlorogenic acid and paclitaxel, whose concentrations remained below the LLOQ (0.01 ng/mL and 10.0 ng/mL, respectively) in all brain homogenate samples, delineates the functional boundaries of this platform, highlighting fundamental compatibility requirements between component structure and drug properties for successful brain delivery.

These findings collectively shift the paradigm from using PS80 as a poorly defined excipient to leveraging it as a modular toolkit for rational formulation design. Our results firmly establish these minor components as promising candidates for targeted brain delivery systems. To fully realize this potential, future work should focus on elucidating precise BBB interaction mechanisms, evaluating the safety and reversibility of permeability-enhancement, and validating performance in disease-relevant models. Ultimately, this work not only clarifies the functional architecture of a key pharmaceutical excipient but also opens new avenues for precision nanomedicine by harnessing the sophisticated functionalities embedded within complex mixtures.

## 5. Patents

Sun, H.; Tu, J.; Wang, J.; Wang, X.; Li, T.; Bi, Q.; Tang, L. A drug-loaded micelle delivery system formed by polysorbate 80 and its components for penetrating the blood–brain barrier. China Patent ZL201810148906.6, issued 29 September 2023.

## Figures and Tables

**Table 1:** Key physicochemical and pharmacokinetic properties of the model drugs.

Model Drugs	Water Solubility	Molecular Weight (g/mol)	Predicted pKa	Charge at pH 7.4 ^c^	Log P Values	BBB Permeant ^d^	P-gp Substrate ^f^
DiR	Insoluble ^a^	1013.39	3 [40]	Cationic	16.59 ^c^	No ^d,e^	Yes ^d,f^
Donepezil	Slightly Soluble ^a^	379.49	16.78 ^b^	Cationic	4.00 ^c^	Yes [37]	Yes [41]
Nimodipine	Practically insoluble ^a^	418.44	16.96 ^b^	Cationic	2.27 ^c^	No ^d,e^	Yes [38]
Chlorogenic acid	Soluble ^a^	354.31	3.33 ^b^	Anionic	−0.39 ^c^	No ^d,e^	No ^d,f^
Paclitaxel	Insoluble ^a^	853.92	11.9 ^b^	Neutral	3.52 ^c^	No ^d,e^	Yes [39]

^a^ descriptive solubility terms are based on the USP classification. ^b^ predicted pKa values were obtained from the DrugBank database [42]. ^c^ charge at pH 7.4 was derived from the predicted pKa. ^d^ data predicted from SwissADME [43,44]. ^e^ BBB permeant: The prediction is based on the BOILED-Egg model, which utilizes the molecule’s lipophilicity and topological polar surface area to predict its passive brain penetration. A molecule is predicted as ‘Yes’ if it is located within the BBB region of the model. ^f^ P-gp substrate: The prediction is generated by a Support Vector Machine model trained on a dataset of known P-gp substrates and non-substrates.

**Table 2:** Particle size (unit: nm) and PDI of drug-loaded formulations determined by DLS. Data depicted as mean (*n* = 3).

Minor Component	DiR		Donepezil		Nimodipine		Chlorogenic Acid		Paclitaxel	
	Particle Size	PDI	Particle Size	PDI	Particle Size	PDI	Particle Size	PDI	Particle Size	PDI
PEG/PS/PI	492	0.01	1003	0.9	497	0.6	393	0.5	17,830	0.9
PSM	1277	0.01	312	0.4	210	0.4	324	0.5	4485	1.0
PIM	273	0.2	406	0.5	155	0.3	227	0.3	12,687	0.9
PSD	21	0.3	12	0.2	13	0.3	191	0.3	26,037	1.0

**Table 3:** Zeta potential (unit: mV) of drug-loaded formulations determined by DLS. Data depicted as mean (*n* = 3).

Minor Component	DiR	Donepezil	Nimodipine	Chlorogenic Acid	Paclitaxel
PEG/PS/PI	+8.3	−4.4	−3.3	−1.5	−14.6
PSM	−1.8	−6.2	−1.4	−2.3	−3.3
PIM	−2.7	−9.4	−3.2	−3.8	−5.7
PSD	−2.5	−2.5	−1.4	−3.0	−3.0

**Table 4:** Summary of BBB permeation-enhancement by PS80 minor components.

Model Drugs	PEG/PS/PI Mixture	PSM	PIM	PSD
DiR	Yes	Yes	Yes	Yes
Donepezil	Yes	Yes	Yes	Yes
Nimodipine	No	Yes	No	No
Chlorogenic acid	No	No	No	No
Paclitaxel	No	No	No	No

## Data Availability

The data presented in this study are available on request from the corresponding author.

## Supplementary Materials

https://www.mdpi.com/article/10.3390/pharmaceutics17121572/s1, Table S1: Semi-quantitative analysis of minor-components distribution in PS80 from five different commercial sources.
