# Supplementary material for: Drug-Dependent Enhancement of Blood–Brain Barrier Permeation by Polysorbate 80 Minor Components
Source: Pharmaceutics. 2025 Dec 5;17(12):1572. doi: 10.3390/pharmaceutics17121572 (PMC12736588; doi:10.3390/pharmaceutics17121572)
Supplement: Supplementary file 1 [file pharmaceutics-17-01572-s001.zip › pharmaceutics-3945196-supplementary.pdf]

S1. Materials and Methods

S1.1. Reagents and materials

Polysorbate 80 (PS80) samples were obtained from five different commercial suppliers (designated as Source A through Source E). Tetrahydrofuran (THF) and methanol were of chromatography grade.

S1.2. HPLC systems for PS80 separation

An appropriate amount of PS80 was accurately weighed, dissolved in methanol, and quantitatively diluted to prepare a test solution at a concentration of approximately 1 mg/mL. The analysis was conducted using a high-performance liquid chromatograph (HPLC) system (Agilent 1260, Agilent Technologies, USA) equipped with an evaporative light scattering detector (ELSD) (Agilent 1260 Infinity II, Agilent Technologies, USA). Chromatographic separation was performed on an Agilent Eclipse XDB-C18 column (4.6 × 150 mm, 5 μm). The mobile phase consisted of (A) tetrahydrofuran (THF) and (B) methanol, with a gradient elution as follows: 0–5 min, 100% to 90% B; 5.0–19 min, 90% to 20% B; and 19–22 min, 20% to 100% B; followed by 3 min equilibration at 100% B. The column temperature was maintained at 30°C, with a flow rate of 1.0 mL/min. The injection volume was 20 μL. The ELSD was operated at a drift tube temperature of 100°C, a gain setting of 1, and a nitrogen flow rate of 1.6 L/min.

S2. Results

The relative abundance of each minor component (PEG/PS/PI mixture, PSM, PIM, PSD) was quantified using HPLC-ELSD. Data are presented as the peak area relative percentage (%) of each minor component in the total detected minor component fraction. The dataset is provided in Table S1.

Table S1. Semi-quantitative analysis of minor-components distribution in PS80 from five different commercial sources.

Source	PEG/PS/PI (%)*	PSM (%)*	PIM (%)*	PSD (%)*
A	31	35	16	13
B	43	36	11	5
C	44	40	5	4
D	15	33	5	28
E	25	34	16	15

* Due to the non-linear response of the ELSD detector, the peak area percentages are presented as a semi-quantitative measure for comparative purposes, rather than as absolute quantitative values. This approach is sufficiently robust to reveal the substantial source-to-source variability in minor component distribution.
